# Role of chemokine C-C motif ligand-1 in acute and chronic pulmonary inflammations

**DOI:** 10.1186/s40064-016-2904-z

**Published:** 2016-08-02

**Authors:** Hiroyuki Kishi, Masamichi Sato, Yoko Shibata, Kento Sato, Sumito Inoue, Shuichi Abe, Tomomi Kimura, Michiko Nishiwaki, Keiko Yamauchi, Takako Nemoto, Akira Igarashi, Yoshikane Tokairin, Osamu Nakajima, Isao Kubota

**Affiliations:** 1 Department of Cardiology, Pulmonology, and Nephrology, School of Medicine, Yamagata University, Yamagata City, Yamagata 990-9585 Japan; 2 Research Laboratory for Molecular Genetics, School of Medicine, Yamagata University, Yamagata City, Yamagata Japan

**Keywords:** CCL1, *Mycobacterium bovis*, Granuloma, Endoplasmic reticulum stress, Lipopolysaccharide, Gene targeted mouse

## Abstract

**Background:**

Chemokine C-C motif ligand 1 (CCL1) accumulates C-C motif chemokine receptor 8 positive leukocytes to the inflammatory sites. Single-nucleotide polymorphisms in the chemokine CCL1 gene are associated with exacerbation of chronic obstructive lung disease. However, it is unclear whether CCL1 has immunomodulatory functions during pulmonary inflammation. This study aimed to elucidate this issue using newly generated transgenic mice that express CCL1 in the lungs (SPC-CCL1 mice).

**Methods:**

To evaluate the phenotypes of these mice, lung section and bronchoalveolar lavage (BAL) fluid analyses were performed. We intratracheally administered lipopolysaccharide (LPS) or *Mycobacterium bovis* as a model of acute or chronic lung inflammation, respectively.

**Results:**

No histological differences were observed between lung tissue from SPC-CCL1 Tg and wild-type mice in the resting condition and after LPS administration. In the resting condition, the total BAL cell concentration was lower in SPC-CCL1 Tg mice than in wild-type mice (*P* = 0.0097). Flow cytometric analyses showed that SPC-CCL1 Tg mice had fewer F4/80-positive cells than wild-type mice (*P* = 0.0278). After intratracheal LPS administration, CCL1 overexpression changed neither the total numbers nor population of BAL cells. After mycobacterial administration, pulmonary granuloma formation was significantly enhanced. The degree of Immunostaining for endoplasmic reticulum to nucleus signaling 1, a molecule associated with granuloma formation and endoplasmic reticulum stress, was significantly enhanced in the granuloma regions of SPC-CCL1 mice treated with *Mycobacterium*, compared to those of wild-type mice.

**Conclusions:**

CCL1 overexpression in the lungs did not change the acute inflammatory response induced by LPS, but enhanced granuloma formation after mycobacterial treatment, possibly through enhancing endoplasmic reticulum stress.

## Background

Chemokines constitute a family of mediators of inflammation and immunity, which are specifically involved in the regulation of immune defense and in immune system housekeeping (Baggiolini 2001; Teran 2000). Chemokine C-C motif ligand 1 (CCL1) is one of the genes that reside in a CCL subfamily chemokine gene cluster on chromosome 17q11.2-q12 in humans (Rollins 1997). CCL1 is known to be produced by activated monocytes/macrophages, T lymphocytes (Th1, Th2, Tc), and endothelial cells (Rollins 1997; Tiffany et al. 1997; Wilson et al. 1988). It mainly acts as a potent chemoattractant for monocytes/macrophages, lymphocytes, and neutrophils and is thought to play a major role in inflammatory processes (Luo et al. 1996; Miller and Krangel 1992; Rollins 1997). Furthermore, C-C motif chemokine receptor 8 (CCR8), a receptor of CCL1, is known to be expressed in monocytes/macrophages, Th2, and regulatory T lymphocytes (Treg) (Rollins 1997; Trebst et al. 2003; Zingoni et al. 1998).

Prior to this study, we demonstrated that single-nucleotide polymorphisms (SNPs) in the CCL1 gene are associated with chronic obstructive pulmonary disease (COPD) exacerbation (Takabatake et al. 2006), that is mainly caused by respiratory tract infection and induces acute worsening of symptoms from the usual stable state (Hurst et al. 2006; Patel et al. 2002; Wilkinson et al. 2006). Carriage of the risk allele for this SNP (National Center for Biotechnology Information SNP: rs2282691: A allele) predisposes patients with COPD to exacerbation. In contrast, carriage of another allele (the T allele) is associated with relative protection from exacerbation (Takabatake et al. 2006). Because this SNP is located in the promoter region of the CCL1 gene, where the C/EBPβ transcription factor binds, this phenomenon appears to be determined by differences in affinity between C/EBPβ and the DNA motif (Takabatake et al. 2006). C/EBPβ is a key transcription factor for acute-phase molecules in response to acute lung injury or infection, and the cardinal role of C/EBPβ is to up-regulate a set of C/EBPβ-regulated genes, including the CCL1 gene, leading to the subsequent activation of the innate immune system and inflammatory response (Didon et al. 2005; Poli 1998). CCL1 has been suggested to play a key role in host defense mechanisms, and up-regulation of CCL1 may defend against infections.

Although the role of CCL1 are investigated, it is still unclear how CCL1 modulates acute and chronic inflammations in the lung. Therefore, we sought to clarify the roles of CCL1 in the lung using newly engineered transgenic (Tg) mice (SPC-CCL1 Tg mice). We evaluated the phenotype of these mice and the in vivo effects of CCL1 overexpression on acute and chronic lung inflammation.

## Methods

### Construction of the SPC-CCL1 plasmid vector

The 3.7 hSP-C/SV-40 plasmid used in this study was kindly provided by Jeffrey A. Whitsett, (Children’s Hospital Medical Center, Cincinnati, Ohio, USA) (Glasser et al. 1988, 1991). This plasmid is composed of a 3.7-kb flanking sequence of the human SP-C promoter and the SV-40 small T intron as a polyadenylation signal (Geraci et al. 1999; Korfhagen et al. 1990). This construct enables specific expression of transgenes in the distal lung epithelium and alveoli (Geraci et al. 1999; Glasser et al. 1991).

**Fig. 1:**
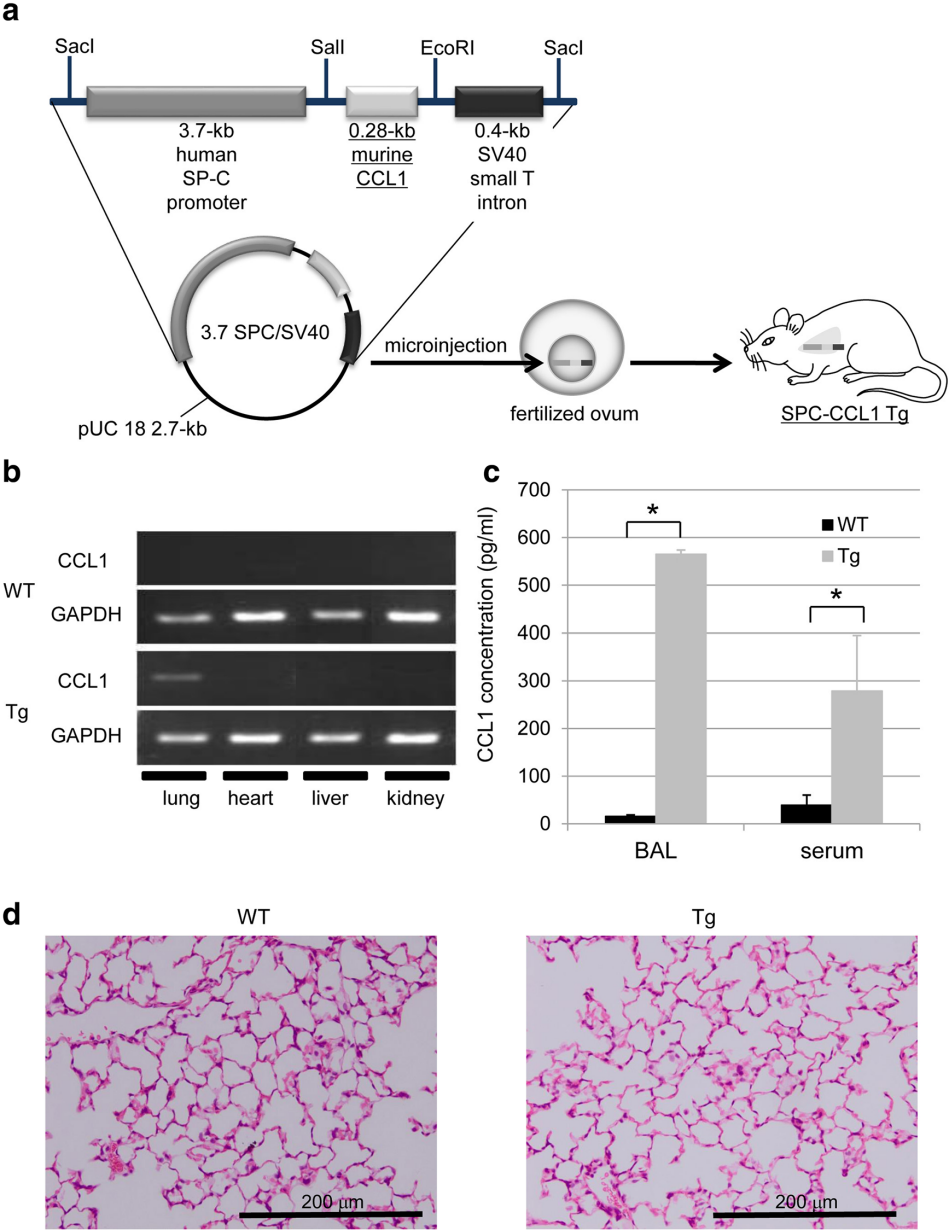
Generation of the CCL1 transgenic (Tg) mice using the surfactant protein C (SP-C) promoter. **a** The plasmid was composed of the 3.7-kb human SP-C promoter and the SV-40 small T intron. The murine CCL1 cDNA was cloned into the *Sal*I*/Eco*RI restriction site, creating the *SPC*-*CCL1* cDNA fusion gene. The resultant plasmid was digested with *Sac*I to generate 4.4-kb (*SPC*-*CCL1*) linear fragments, which were microinjected into the pronuclei of fertilized C57BL/6 mouse eggs and implanted into a foster mother mouse to induce a pseudopregnancy. **b** Gene expression of SPC-CCL1 and GAPDH evaluated by RT-PCR. CCL1 gene expression was confirmed by RT-PCR using specific primers. CCL1 gene expression was upregulated in the lungs of SPC-CCL1 Tg mice, but negative in those of wild-type (WT) mice. **c** Comparison of CCL1 levels in BAL fluid between Tg and WT mice. CCL1 concentrations were measured by ELISA. The levels of CCL1 in BAL fluid and serum were elevated in SPC-CCL1 Tg mice (*gray bar*) compared to WT mice (*black bar*). *P value <0.01. **d** Histological findings for the lungs (hematoxylin and eosin stain, ×200). No morphological changes were observed in the lungs of SPC-CCL1 Tg mice (*right*), compared to WT mice (*left*)

The murine CCL1 mRNA sequence was ascertained by searching the NCBI library (NM_011329) and the Ensembl genome browser (ENSMUSG00000020702). The mRNA of murine CCL1 consists of 514 bp, including the untranslated region and the coding sequence. We prepared an insert genome sequence comprising 283 bp, including the entire coding sequence, and added restriction enzyme sites, *Sal*I (GTCGAC) and *Eco*RI (GAATTC), before and after the sequence. Invitrogen™ assisted in sequence manufacture. The murine CCL1 cDNA was cloned into the *Sal*I/*Eco*RI restriction site of the 3.7 hSP-C/SV-40 plasmid, creating the *SPC*-*CCL1* cDNA fusion gene (Fig. 1a). The proper orientation of the plasmid was confirmed using an ABI PRISM 3130 Genetic Analyzer, and polymerase chain reaction (PCR) using Platinum Taq DNA polymerase (Invitrogen, Carlsbad, CA, USA) using specific primers. We designed specific primers across the sequence of CCL1 and SV40 DNA (forward: TGT CCC TCT CCT ACG GAC AC, reverse: CCA CAG AAG TAA GGT TCC TTC ACA). These primers were also used to verify the genomic DNA sequence of the transgene in vivo.

### Generation of SPC-CCL1 Tg mice

The resultant plasmid was digested with *Sac*I to generate 2.7-kb (pUC 18 DNA) and 4.4-kb (*SPC*-*CCL1*) linear fragments, which were microinjected into the pronuclei of fertilized C57BL/6 mouse eggs and implanted into a foster mother mouse to induce a pseudopregnancy. We commissioned the Research Laboratory for Molecular Genetics (Faculty of Medicine, Yamagata University) to generate transgenic mice.

The rooms were kept free of any pathogens. All mice were handled according to the guide for the care and use of laboratory animals of Yamagata University, and the study protocol was approved by the Animal Subjects Committee of Yamagata University (23068, 24068, 25068, 26068, and 27068). The study was also performed in accordance with the Declaration of Helsinki.

### Histological analysis

Mice were anesthetized by intraperitoneal injection of 10 % pentobarbital (100 mg/kg body weight), and were sacrificed for collection of blood and excision of organs. The lungs were inflated intratracheally under constant positive pressure at 25 cm H_2_O with 4 % paraformaldehyde for fixation and resected to prepare paraffin-embedded lung blocks for morphological examinations (Hirama et al. 2007). Other tissues were also fixed with 4 % paraformaldehyde, and embedded into paraffin. Tissue sections were cut at a 3-μm thickness and placed on glass slides, subsequently stained with hematoxylin and eosin, and examined using a microscope (BX50F4; Olympus, Tokyo, Japan).

### Extraction of RNA and reverse transcription (RT)-PCR

Total RNA was extracted from lung, heart, liver, and kidney tissues using TRIzol reagent (Invitrogen, Carlsbad, CA, USA) according to the manufacturer’s protocol. RT was performed using SuperScript III reverse transcriptase (Invitrogen, Carlsbad, CA, USA) and gene expression was assessed by RT-PCR. The sequences of the specific primers were as follows: CCL1; identical to the primers described above, GAPDH; forward, CTT CAC CAC CAT GGA GAA GGC; reverse, GGC ATG GAC TGT GGT CAT GAG, CCR8; forward, TGG TCT TCC TGC CTC GAT GGA; reverse, CAG GCT GTT CCC CAG AAG GCC CA. PCR products were electrophoresed in 2.0 % agarose gels containing ethidium bromide, and visualized digitally with an ultraviolet illuminator (AB1500 Printgraph and AE 6905H Image Saver HR; ATTO Bioscience, Tokyo, Japan) (Machiya et al. 2007).

### Preparation of blood

Whole blood was drawn by direct puncture of the inferior vena cava in mice, which had been deeply anesthetized with intraperitoneal 10 % pentobarbital (100 mg/kg body weight). Blood samples were used for three different purposes; whole blood cell counts, serum collection, and preparation of white blood cell suspensions. For whole blood cell counts, blood was collected into tubes containing ethylenediaminetetracetic acid (EDTA), and was analyzed using a whole blood counter (Celltacα; Nihon Kohden, Tokyo, Japan). For serum collection, blood was collected into tubes without EDTA, and thereafter individual serum samples were separated from the clotted blood by centrifugation and stored at −80 °C until the assays were performed. For preparation of white blood cell suspensions, whole blood was collected into tubes containing EDTA, blood cell suspensions were prepared after hemolysis of erythrocytes by distilled water, and the resultant suspensions were used for flow cytometry.

### Bronchoalveolar Lavage (BAL) procedure

Mice were anesthetized and a 20-gauge metallic catheter was inserted into the trachea. BAL was performed by administering 1 ml of Hanks’ balanced salt solution (HBSS) containing 0.5 mM EDTA 3 times with a syringe. These manipulations were performed gently to avoid artificial lung injury. Total BAL cell numbers were counted under a light microscope using a hemocytometer. Fractions of the BAL cells were attached to glass slides by cytospinning at 1000 rpm for 5 min (Shandon Cytospin 2 Cytocentrifuge; Thermo, Waltham, MA, USA), and stained with a Diff-Quick solution (International Reagents Corp., Kobe, Japan) for differential cell counts (Hirama et al. 2007). The remaining cells were divided from the BAL fluid by centrifugation at 1200 rpm for 5 min, supernatants were stored at −80 °C until evaluation by ELISA, and cell suspensions were used for flow cytometry.

### Biochemical analysis of BAL fluid and serum

Murine CCL1, interleukin-6 and tumor necrosis factor-alpha in the BAL fluid and serum were measured by ELISA (Quantikine; R&D Systems, Minneapolis, MN, USA), according to the manufacturer’s protocols.

### Flow cytometry

Cell surface markers of BAL cells (F4/80, CD11b, Gr-1) from WT or SPC-CCL1 Tg mice were analyzed by flow cytometry. Cells were resuspended in fluorescence-activated cell sorter (FACS) buffer (PBS supplemented with 0.2 % BSA, 0.01 % sodium azide). BAL cells were stained with FITC-labeled F4/80 antibody (AbD; Serotec, Kidlington, UK); PerCP-labeled CD11b antibody (BD Pharmingen, CA, USA), PE-labeled Gr1 antibody (BD Pharmingen, CA, USA), or an isotype control (BD Pharmingen, CA, USA) at a concentration of 10 µg/ml at 4 °C for 30 min in the dark. Stained cells were washed once in FACS buffer and subjected to flow cytometric analysis using a FACS Canto II (BD Biosciences, CA, USA) (Sato-Nishiwaki et al. 2013). Data were analyzed using DIVA software (BD Bioimaging Systems, MD, USA) and FlowJo 7.6.1 (Tree Ster, Inc., Ashland, OR, USA).

### Acute lung inflammation model

Mice were anesthetized by intraperitoneal injection of 10 % pentobarbital (80 mg/kg body weight), and the trachea was intubated with a 22-gauge cannula. Lipopolysaccharide (LPS, 2 mg/kg body weight) derived from *Escherichia coli* O55: B5 (SIGMA-ALDRICH. St. Louis, MO, USA) in isotonic sodium chloride solution was intratracheally administered via a cannula at a determinate amount (50 µl/mouse) (Chen et al. 2013). Mice were sacrificed after 6, 24, or 72 h from administration, and were evaluated for histological findings of lung, BAL cell count, and cell differentials.

### Chronic lung injury model

Bacille de Calmette et Guérin (BCG, 6 × 10^6^/mouse) derived from *Mycobacterium bovis* (Tokyo 172 strain) (Japan BCG Laboratory. Tokyo, Japan) in isotonic sodium chloride solution was intratracheally administered via a cannula at a determinate amount (100 µl/mouse) to anesthetized mice (Fulton et al. 2000; Tokairin et al. 2008). Mice were sacrificed after 28 days from administration for the preparation of pathological samples and the isolation of total RNA from the lungs. In the pathological analyses, three digitized tissue images were randomly taken using a microscope system at the 10× magnification from all lobes of each mouse, and the total number of granulomas was counted.

### Immunohistochemistry

Paraffin-embedded sections (3 μm thick) were cut and placed on glass slides. For detection of immunoglobulin (Ig), sections were incubated with antibodies to horseradish peroxidase-labeled mouse IgG (Promega Corp., Madison, WI, USA) at 1:250 dilution. For detection of endoplasmic reticulum to nucleus signaling (Ern)-1, sections were incubated with antibodies to phosphorylated Ern1, also known as inositol-requiring enzyme-1a (IRE1a) (Novus Biologicals, Littleton, CO, USA) at 1:1000 dilution, followed by horseradish peroxidase-labeled anti-rabbit IgG (Promega Corp., Madison, WI, USA) at 1:200 dilution. Color development was performed using the DAKO Liquid DAB + Substrate Chromogen System (Dako, Glostrup, Denmark). Immunostaining of all sections was performed simultaneously, to avoid variation due to differences in staining conditions. Cell nuclei were stained with hematoxylin.

To evaluate the positivity of IgG and Ern1, intensities of IgG and Ern1 immunohistochemistry in each granuloma were scored according to the scoring system described in the previous literature with some modification (Serth et al. 1995): 0, no staining at all; 1, staining in <5 % of cells; 2, staining in 5–10 % of cells; 3, staining in 11–39 % of cells; 4, staining in >40 % of cells. This assessment was performed in the twenty granulomas in each mice, and the mean was used for the representative value for a mouse.

### Evaluation of bacterial burden after inoculation of BCG

As described above, BCG (6 × 10^6^/mouse) was intratracheally administrated to mice. DNA was isolated from removed lungs using Allprep DNA/RNA Mini Kit (QIAGEN). MBP70, a BCG genomic DNA, and Ywhaz, a genomic mouse DNA, was amplified using real-time PCR system (Kimura et al. 2012). The sequences of the specific primers were as follows: MBP70; forward, ACC AGC ATC CTG ACC TAC CAC; reverse, CTT GAG GCT GTT ACC CTG ACC, and Ywhaz4; forward, GTA AAG TGC AGC CCA CGA CA; reverse, AGG AAG CGG CCT TAG ACC TT. The primers for Ywhaz were designed from the sequence between intron 1 and 2. The ratio of the PCR-amplification level in MBP to that in Ywhaz were considered as bacterial burden of BCG (Cousins et al. 1992; de Assuncao et al. 2014). Data were expressed as fold change compared to wildtype mice.

### Statistical analysis

The results are expressed as mean ± standard deviation (SD). Student’s *t* test was used to evaluate differences in means. The non-parametric Mann–Whitney U test was used to compare variables that were not normally distributed. All statistical analyses were performed using JMP version 9 software (SAS Institute Inc., Cary, NC, USA). Statistical significance was defined as *P* < 0.05.

## Results

### Confirmation of CCL1 mRNA overexpression

Three transgenic founders (lines 6, 8, and 63) were generated, as confirmed by PCR of genomic DNA. CCL1 mRNA expression of SPC-CCL1 Tg mice was confirmed by RT-PCR using specific primers. Transgene expression of CCL1 was detected only in the lungs of SPC-CCL1 Tg mice, and no CCL1 expression was observed in other organs, or in WT mice. Representative images of CCL1 and GAPDH expression in line 63 are shown in Fig. 1b. The levels of CCL1 in BAL fluid and serum were significantly elevated in SPC-CCL1 Tg mice, compared with WT mice (Fig. 1c). The CCL1 concentration in the BAL fluid was lower in the other two lines of transgenic mice than in line 63 (data not shown). Thus, line 63 was used for the following experiments.

### Evaluation of phenotypes

No neonatal death was observed in SPC-CCL1 mice, and the survival rate was similar to that of WT mice (data not shown). The general appearance of SPC-CCL1 Tg mice was not obviously different from that of WT mice (data not shown). No morphological differences in lung tissue were observed between SPC-CCL1 Tg mice and WT mice (Fig. 1d). Histological findings for other tissues (heart, thymus, liver, spleen, pancreas, kidney, adrenal gland, and skin) did not differ significantly between SPC-CCL1 Tg mice and WT mice (data not shown).

There were no significant differences in organ weight between SPC-CCL1 Tg mice and WT mice (Table 1). In the whole blood counts, SPC-CCL1 Tg mice and WT mice did not differ significantly in numbers of white blood cells or red blood cells, but the platelet count was significantly higher in SPC-CCL1 Tg mice than in WT mice (Table 1).

**Table 1:** Comparisons of organ weight/blood count between wild-type (WT) and SP-C CCL-1 transgenic (Tg) mice at resting condition

	WT (n = 4)	Tg (n = 4)	*P* value
BW (g)	25.7 ± 2.9	24.5 ± 0.8	0.3865
Lung (g)	0.119 ± 0.009	0.118 ± 0.009	0.7728
Heart (g)	0.113 ± 0.006	0.114 ± 0.010	0.5637
Liver (g)	1.138 ± 0.102	1.101 ± 0.085	0.5637
Spleen (g)	0.074 ± 0.012	0.070 ± 0.006	0.2482
Kidney (g)	0.279 ± 0.048	0.244 ± 0.023	0.3865
Thymus (g)	0.035 ± 0.003	0.033 ± 0.006	0.5637
WBC (×10^2^/µl)	54.20 ± 16.36	65.40 ± 25.19	0.3472
RBC (×10^4^/µl)	981.6 ± 125.3	845.6 ± 44.8	0.0758
Hemoglobin (g/dl)	15.8 ± 1.8	13.7 ± 0.4	0.0740
Hematocrit (%)	46.6 ± 6.1	43.2 ± 1.7	0.3472
Platelet (×10^4^/µl)	68.6 ± 14.6	88.5 ± 10.5	0.0283

Values are given as the mean ± SD

Mann–Whitney U test

*BW* body weight, *WBC* white blood cell, *RBC* red blood cell

### Analysis of BAL cell numbers and differential cell counts

Concentrations of BAL cells and total cell count were significantly lower in SPC-CCL1 Tg mice than in WT mice (Table 2). Furthermore, the number of macrophages was lower in SPC-CCL1 Tg mice than in WT mice (Table 2).

**Table 2:** Comparison of BAL cells between wild-type (WT) and SP-C CCL-1 transgenic (Tg) mice at resting condition

	WT (n = 16)	Tg (n = 15)	*P* value
BW (g)	25.8 ± 2.0	26.2 ± 2.8	0.6734
Collection volume (ml)	2.93 ± 0.07	2.93 ± 0.07	0.9471
Cell concentrations (×10^4^/ml)	2.00 ± 0.73	1.37 ± 0.52	0.0097
Total cell count (×10^4^)	8.16 ± 2.19	4.31 ± 1.24	0.0112
Macrophages (×10^4^)	7.66 ± 1.33	4.31 ± 1.24	0.0103
Neutrophils (×10^4^)	0.46 ± 0.54	0.00 ± 0.00	0.1349
Lymphocytes (×10^4^)	0.04 ± 0.06	0.00 ± 0.00	0.2688
Eosinophils (×10^4^)	0.00 ± 0.00	0.00 ± 0.00	–

Values are given as the mean ± SD

Mann–Whitney U test

*BW* body weight

### Analysis of surface markers by flow cytometry (FCM)

**Fig. 2:**
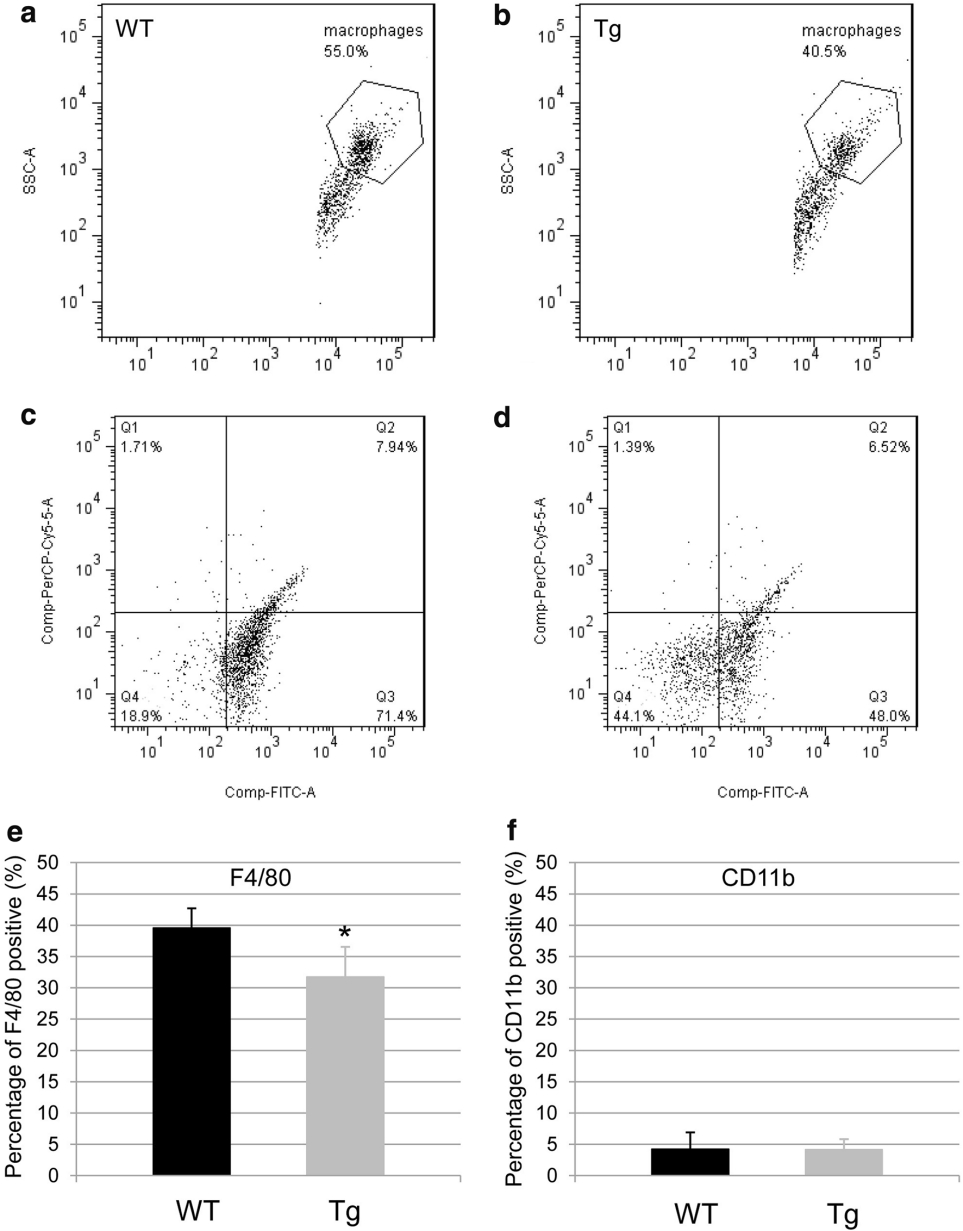
Flow cytometric (FCM) plots of BAL cells and alveolar macrophages. Representative schemas for dot plots for FCM are shown. BAL cells of WT mice (**a**) and SPC-CCL1 Tg mice (**b**) were distributed by forward scatter (FSC) and side scatter (SSC). Cells regarded as alveolar macrophages were enclosed hexagonal form. Alveolar macrophages were decreased in SPC-CCL1 Tg mice compared to WT mice. FSC and SSC were expressed on a log scale, and smaller objects (FSC < 5000) were omitted from analyses as debris. The fraction of macrophages was gated and the surface markers of WT mice and SPC-CCL1 Tg mice were evaluated. The gated fractions of AMs from WT mice (**c**) and SPC-CCL1 Tg mice (**d**) were distributed by FITC-F4/80 and PerCP-CD11b. **e** F4/80-positive cells were decreased in SPC-CCL1 Tg mice (*gray bar*) compared to WT mice (*black bar*). **f** CD11b-positive cells remained unchanged compared to WT mice. *P value <0.05 compared to WT mice

Dot plots of BAL cells by FCM are shown in Fig. 2. BAL cells of WT mice (Fig. 2a) and SPC-CCL1 Tg mice (Fig. 2b) were distributed by forward scatter (FSC) and side scatter (SSC). FSC and SSC were expressed on a log scale, and smaller objects (FSC < 5000) were omitted from analyses as debris. Cells regarded as alveolar macrophages (AMs) were enclosed hexagonal forms, and gated for analysis (Tailleux et al. 2005). The results showed fewer AMs in SPC-CCL1 Tg mice than in WT mice.

Subsequently, the gated fractions of AMs of WT mice and SPC-CCL1 Tg mice were evaluated for expression of surface markers (F4/80 and CD11b) (Fig. 2c, d). Expression of F4/80 on AMs from SPC-CCL1 Tg mice was attenuated, compared with that of WT mice (Fig. 2e, Tg mice: 31.8 ± 4.8 %, WT mice: 39.6 ± 3.1 %, *P* = 0.0278). However, expression of CD11b on AMs from SPC-CCL1 Tg mice remained unchanged, compared with that of WT mice (Fig. 2f, Tg mice: 4.2 ± 1.7 %, WT mice: 4.2 ± 2.7 %, *P* = 0.9168).

### Analysis of acute lung injury model

**Fig. 3:**
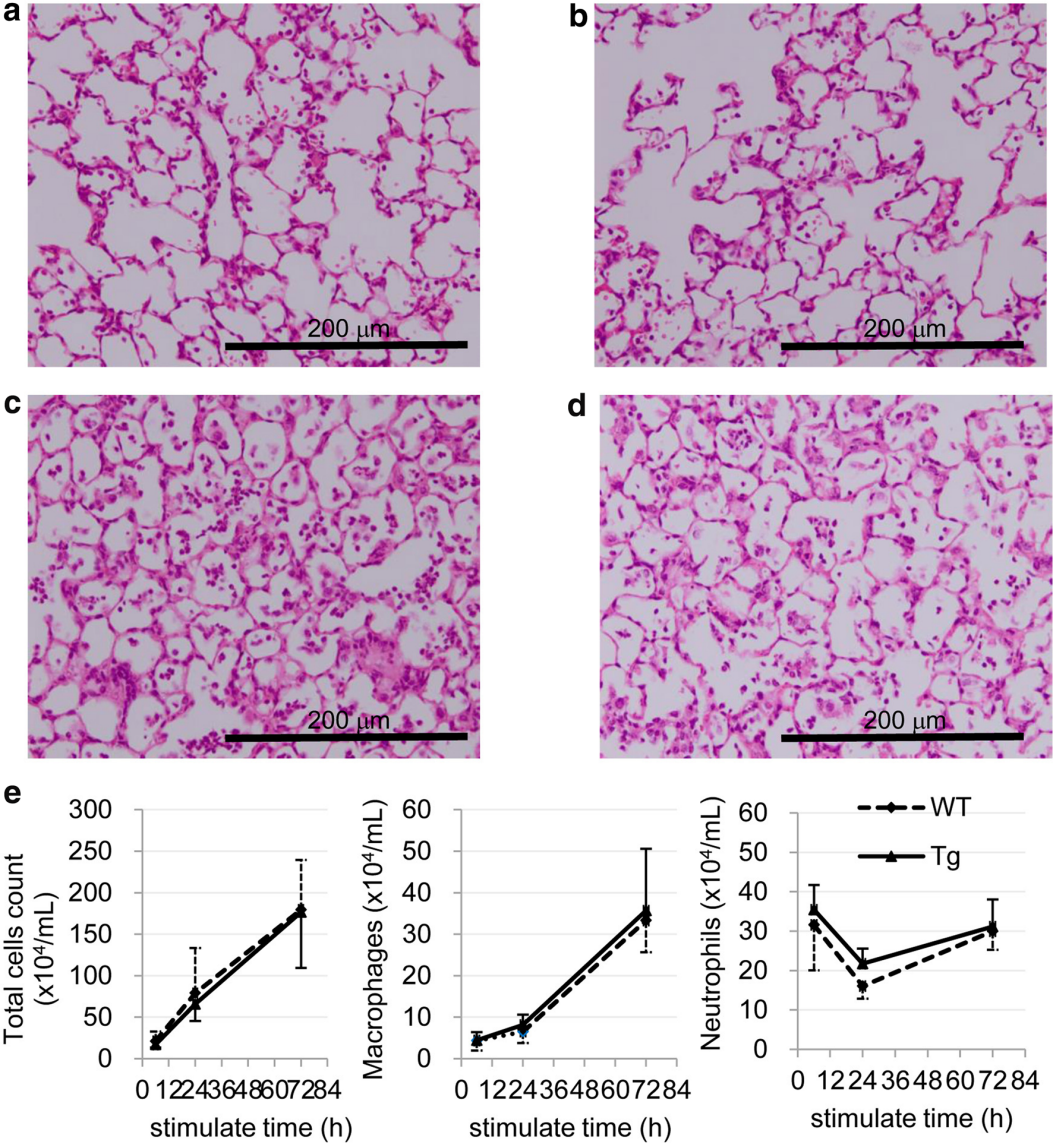
Intratracheal administration of lipopolysaccharide (LPS) into the lungs of wild-type (WT) and SPC-CCL1 transgenic (Tg) mice. To produce a model of acute lung injury, mice were intratracheally administered LPS (2 mg/kg body weight). **a** WT mice, 6 h after administration. Inflammatory cells such as neutrophils had infiltrated the alveoli. **b** SPC-CCL1 Tg mice, 6 h after administration. Infiltration of inflammatory cells was also observed. **c** WT mice, 72 h after administration. The alveoli were filled with inflammatory cells, particularly neutrophils, macrophages, and exudates. **d** SPC-CCL1 Tg mice, 72 h after administration. Histopathological findings did not show significant differences between LPS-treated SPC-CCL1 Tg mice and WT mice. **e** Time course of changes in BAL cells (*left* total cells, *middle* macrophages, *right* neutrophils) 6, 24 and 72 h after LPS administration. CCL1 overexpression changed neither the total number nor population of BAL cells

Mice were intratracheally administered LPS (2 mg/kg body weight) and sacrificed at 6, 24, or 72 h after administration. At 6 h after administration, inflammatory cells, such as neutrophils, had infiltrated the alveoli of both WT (Fig. 3a) and SPC-CCL1 Tg mice (Fig. 3b). Infiltration of inflammatory cells was almost equal between the two groups. At 72 h after administration, the alveoli were filled with inflammatory cells, particularly neutrophils, macrophages, and exudates. Histopathological findings did not show significant differences between LPS-treated SPC-CCL1 Tg mice (Fig. 3d) and WT mice (Fig. 3c).

Figure 3e shows a time course of change in BAL cells after LPS administration. However, CCL1 overexpression changed neither the total numbers nor population of BAL cells (Fig. 3e). In addition, the levels of interleukin-6 and tumor necrosis factor-alpha in BAL fluid after LPS intratracheal administration were not different in between WT and Tg mice (Table 3).

**Table 3:** Comparison of Interleukin-6 (IL-6) and tumor necrosis factor-alpha (TNF-alpha) in bronchoalveolar lavage fluid after intratracheal administration of lipopolysaccharide between wild-type (WT) and SP-C CCL-1 transgenic (Tg) mice

	Time point (h)	WT (n = 5)	Tg (n = 5)	*P*
IL-6	0	18.6 ± 6.2	19.8 ± 6.4	0.77
	6	254.2 ± 90.7	385.6 ± 107.8	0.63
	24	1336.0 ± 500.3	1500.6 ± 331.2	0.56
	72	915.6 ± 377.9	1141.0 ± 423.0	0.4
TNF alpha	0	18.0 ± 7.6	22.0 ± 7.5	0.43
	6	563.2 ± 198.1	728.6 ± 218.6	0.26
	24	1576.4 ± 546.8	1468.8 ± 589.9	0.77
	72	315.8 ± 126.6	417.2 ± 174.8	0.32

Values are given as the mean ± SD

Mann–Whitney U test

### Analysis of chronic lung inflammation model

**Fig. 4:**
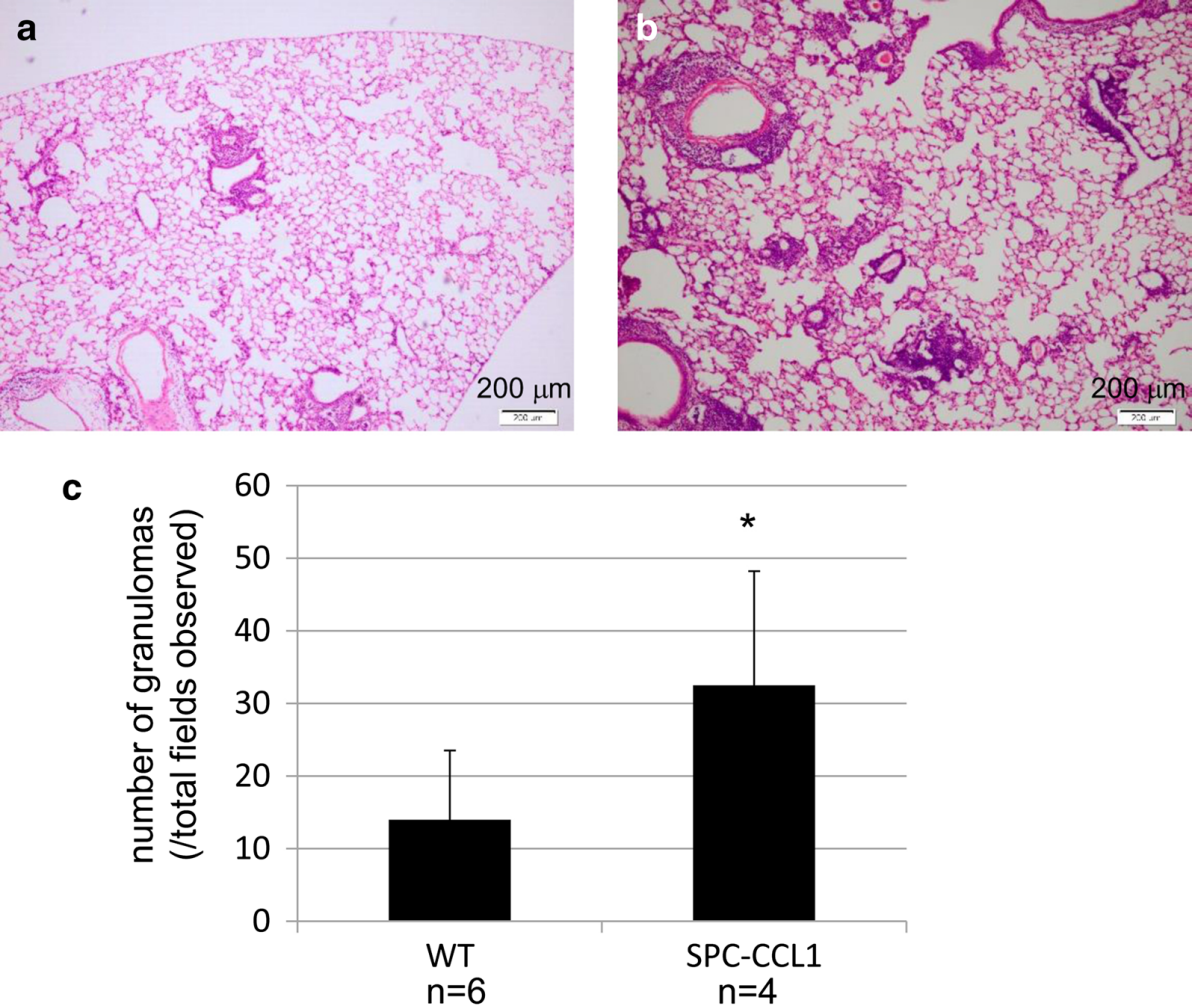
Intratracheal administration of *Mycobacterium* into the lungs of wild-type (WT) and SPC-CCL1 transgenic (Tg) mice. To produce a model of chronic lung inflammation, mice were intratracheally administered Bacille de Calmette et Guérin (BCG). **a** Representative lung image of WT mice, 28 days after administration. **b** Representative lung image of SPC-CCL1 Tg mice, 28 days after administration. Enhanced granuloma formation was observed in the lungs of Tg mice, compared to WT mice **c**. *P value <0.05 compared to WT mice

The lungs of WT and Tg mice were infected with *M. bovis* (BCG) to produce chronic pulmonary inflammation. Compared to the lungs of BCG-infected WT mice (Fig. 4a), granuloma formation was increased in Tg mice (Fig. 4b). Quantification was performed as described in the “Methods”, and the difference was confirmed (Fig. 4c). There were no significant bacterial burdens between WT and Tg mice (1.13 ± 0.60 fold vs wild type, *P* = 0.74, n = 4 in each group).

**Fig. 5:**
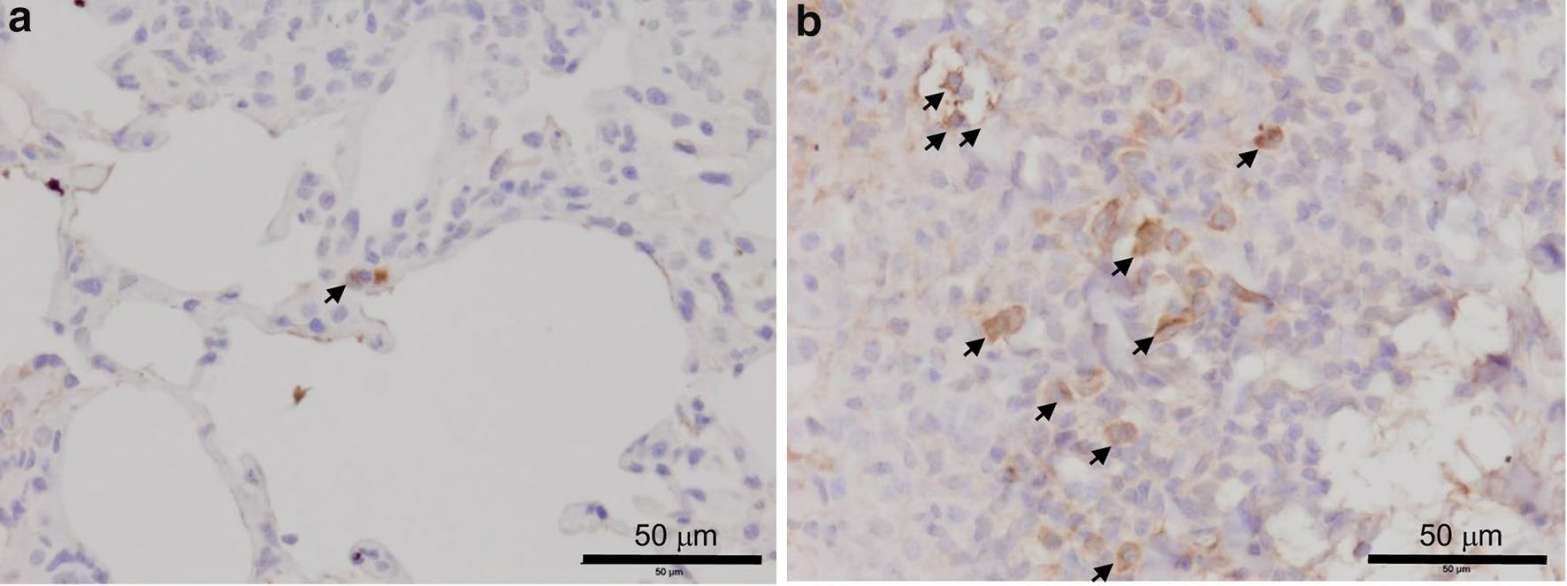
Immunohistochemical staining using an antibody to immunoglobulin G in the lungs of BCG-treated wild-type (WT) and SP-C CCL-1 transgenic (Tg) mice. Pulmonary immunoglobulin G was immunohistochemically stained in the lungs of WT (**a**, n = 6) and Tg mice (**b**, n = 4) 21 days after BCG administration, as described in the “Methods”. Representative images are shown. *Black arrows* indicate positive cells. Original magnification ×800

Antibody-mediated immunity is reported to be important for mycobacterial infection (Chan et al. 2014). Immunohistochemical analysis using an antibody against IgG revealed more IgG-positive cells in the granulomatous lesions of BCG-treated Tg mice than in those of WT mice [Fig. 5a, b: IgG immunostaining score: WT, 1.3 ± 0.6 (n = 6); Tg 2.7 ± 0.6 (n = 4); *P* < 0.05 Mann–Whitney U test].

### Enhanced endoplasmic reticulum (ER) stress in SPC-CCL1 transgenic mice after mycobacterial infection

**Fig. 6:**
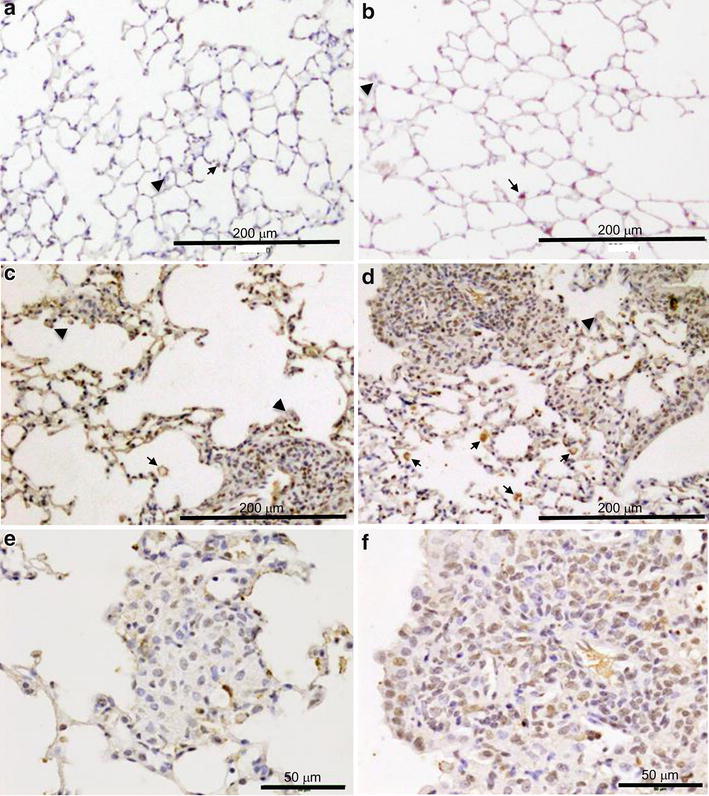
Immunohistochemical staining using an antibody to phosphorylated endoplasmic reticulum to nucleus signaling 1 (Ern1) in the lungs of BCG-treated wild-type (WT) and SP-C CCL-1 transgenic (Tg) mice. Phosphorylated Ern1 was immunohistochemically stained in the lungs of WT and Tg mice 21 days after BCG administration, as described in the “Methods”. *Black arrows* indicate phosphorylated Ern1-positive alveolar macrophages. *Black arrowheads* indicate phosphorylated Ern1-negative alveolar macrophages. **a** WT untreated, **b** Tg untreated, **c** WT BCG-treated; **d** Tg BCG-treated, **e** WT BCG-treated, **f** Tg BCG-treated. **a**–**d** original magnification ×200, **e**, **f** original magnification ×800

Recently, the involvement of ER stress was reported in the pathogenesis of granuloma formation induced by *Mycobacterium* (Seimon et al. 2010). Immunohistochemical analysis showed that alveolar macrophages in the lungs of Tg mice treated with BCG were more positive for phosphorylated Ern1 than those of WT mice (Fig. 6c, d). The nuclei in granuloma cells of BCG-treated Tg mice were more positive for phosphorylated Ern1 than those of WT mice [Fig. 6e, f: Ern1 immunostaining score: WT, 3.1 ± 0.1 (n = 6); Tg 3.7 ± 0.1 (n = 4); *P* < 0.05 Mann–Whitney U test].

## Discussion

In this study, we generated Tg mice that overexpress CCL1 specifically in the lungs (Fig. 1), and the activity of the transgene was verified by the high concentrations of CCL1 in the BAL fluid and serum (Fig. 1c). The receptor for CCL1, CCR8, is not expressed on pulmonary epithelium, endothelium, or fibroblast. Therefore, pulmonary expression using SP-C promoter thought to be usable to evaluate the immune reaction of CCR8-positive leukocytes to CCL1, although the major source of CCL1 is different between CCL1 Tg and WT mice. Because we did not detect the gene expression of CCL1 in other organs of CCL1 mice (Fig. 1b), the elevation of CCL1 seems to be due to spill-over of lung CCL1. However, the number of BAL cells, particularly the number of AMs, was significantly lower in these Tg mice than in WT mice (Table 2). In addition, F4/80, a macrophage differentiation marker, was significantly reduced on AMs recovered from the BAL from resting-state Tg mice compared to WT mice (Fig. 2). In the model of acute lung inflammation, the accumulation of inflammatory cells did not significantly differ between the lungs of WT and Tg mice (Fig. 3). Notably, in the model of chronic inflammation, formation of granulomas after BCG administration was significantly enhanced in the lungs of Tg mice compared to WT mice (Fig. 4). Immunohistochemical analysis showed the higher levels of phosphorylated Ern1 in the alveolar macrophages and nuclei of granuloma cells in BCG-treated CCL1 Tg mice than in WT mice (Fig. 6).

CCL1 mainly acts as a potent chemoattractant for monocytes/macrophages, lymphocytes, and neutrophils. Therefore, the accumulation of the leukocytes to the lungs was expected in the CCL1 Tg mice. However, against this prediction, the total cells and alveolar macrophages recovered from BAL were significantly reduced in SPC-CCL1 Tg mice. The mechanism for this phenomenon has not been elucidated. In the lungs of these Tg mice, CCL1 is constitutively expressed, and a high level of CCL1 is maintained. Thus, in these mice, lung cells are chronically exposed to a high level of CCL1. Although acute chemotactic effects of CCL1 on inflammatory cells have been shown by several investigators (Doyle and Murphy 1999; Luo et al. 1994; Miller and Krangel 1992; Tiffany et al. 1997), the chronic effects of CCL1 have not been investigated. This difference in exposure of the cells to CCL1, acute or chronic, may alter the cellular response to this chemokine. Furthermore, chronic exposure to CCL1 might alter the expression of its receptor, CCR8, or the status of downstream molecules associated with CCL1-CCR8 signal transduction. However, we could not detect the expression of CCR8 in lung tissue or BAL cells of both CCL1 Tg and WT mice using RT-PCR methods, probably due to the low expression of CCR8 in lung tissue and alveolar macrophages, although CCR8 expression was positive in thymus of WT and CCL1 Tg mice (data not shown). Because we could not find any literatures describing the expression of CCR8 in the murine alveolar macrophages, the expression of CCR8 may be lost during the process to terminal differentiation of alveolar macrophages in mice. CCR8 expression in circulating monocyte should be examined using flow cytometry technique in the future in CCL1 Tg mice.

Total BAL cells, especially alveolar macrophages, and expression of the surface marker, F4/80, in AMs of Tg mice was significantly reduced (Table 2; Fig. 2e). F4/80 has been established as a specific cell surface marker for murine macrophages (Austyn and Gordon 1981; Morris et al. 1991), and is known to be a member of the epidermal growth factor-seven-transmembrane-domain family. As the precursors of tissue macrophages, blood monocytes express less F4/80 than mature macrophages (Gordon et al. 1992). CD11b is expressed on the surface of monocytes, macrophages, NK cells, and granulocytes. AMs express less CD11b than their monocytic precursors (Senft et al. 2007). In the present study, F4/80 expression was reduced in the AMs of SPC-CCL1 Tg mice compared to those of WT mice. However, the mechanisms for decreasing numbers and surface markers of alveolar macrophages in CCL1 Tg mice was unclear. The alveolar macrophage numbers and functions are known to be regulated by hematopoietic growth factors such as M-CSF and GM-CSF (Shibata et al. 2001a, b). CCL1 may modulate the expression of these growth factors or their receptors. In future work, we will need to evaluate the levels of M-CSF, GM-CSF, receptors for M-CSF and GM-CSF, and the transcription factors that regulate the differentiation and functions of macrophages, such as PU.1, MafB, and interferon regulatory factor-8 (Sato-Nishiwaki et al. 2013; Shibata et al. 2001a; Tamura et al. 2000).

In the present study, CCL1 overexpression did not alter accumulation of inflammatory cells after LPS stimulation (Fig. 3), although the baseline number of alveolar macrophages were significantly reduced (Table 2). This result was consistent with a previous report that demonstrated a lack of induction of CCL1 by stimulation with LPS, IFN-γ, interleukin-1beta (IL-1β), tumor necrosis factor alpha (TNFα), IL-4, IL-13, IL-10, IL-6, IL-18, and combinations thereof in human monocytes, induction of CCL1 in monocytes, and the requirement for engagement of FcγR II following exposure to IL-1β or LPS (Sironi et al. 2006). CCL1 did not play major roles in this acute lung inflammation model.

CCL1 is suggested to be involved in the pathogenesis of mycobacterial infection, one of the most important chronic inflammation in the lung (Yu et al. 2012). Increase of CCL1 was reported in the animal experimental model of granuloma by Mycobacterium (Chiu et al. 2003). Seimon et al. demonstrated induction of ER stress and upregulation of Ern1 in granuloma cells by administration of *M. tuberculosis* to mice (Seimon et al. 2010). In the present study, we demonstrated the enhanced phosphorylation of Ern1 in the granulomas of CCL1 Tg mice (Fig. 6). To the best of our knowledge, there are no studies that have investigated the relationship between CCL1 and Ern1. However, it has been reported that overexpression of CCL2, another chemokine, induces ER stress in a preadipocyte cell line. Inhibition of ER stress by taurursodeoxycholate or knockdown of Ern1 inhibited CCL2-induced protein caused autophagy and adipogenesis (Younce and Kolattukudy 2012). Increase of reactive oxygen species (ROS) is suggested to be involved in one of the mechanism governing CCL2-induced ER stress (Younce and Kolattukudy 2012). Because CCL1 also induces reactive oxygen in the presence of LPS in a dose-dependent manner (Reimer et al. 2011), it is possible that the increase of ROS enhances the ER stress in CCL1 Tg mice. The mechanism governing the upregulation of Ern1 in the BCG-treated Tg mice needs to be elucidated with the precise in vitro experiments in the future.

Ern1 possesses an intrinsic kinase activity and an endoribonuclease activity, and it is important in altering gene expression in response to endoplasmic reticulum-based stress signals. Although it has not been elucidated whether ER stress plays a beneficial role in the host response to infection, ER stress interacts with and regulates signaling intermediates involved in the activation of the innate and adaptive immune responses (Muralidharan and Mandrekar 2013). It has been reported that Ern1 is necessary for the development of adaptive immune B cells, terminally differentiated plasma cells (Iwakoshi et al. 2003; Zhang et al. 2005). As shown in Fig. 5, immunoglobulin positive cells were increased in the granuloma of CCL1 Tg mice. Thus, enhanced phosphorylation of Ern1 may be essential for the alteration of immune status observed in the granuloma cells of BCG-treated CCL1 Tg mice.

In the chronic lung inflammation model using intratracheal administration of BCG, granuloma formation was significantly increased in Tg mice. Since bacterial burden after BCG inoculation was not significantly different between two groups (text in the “Results” section), this enhanced formation of granuloma suggests the involvement of the hyper-response to biotic stimulus in CCL1 Tg mice, not due to reduced bacterial killing capacity.

In conclusion, the present study demonstrated that CCL1 overexpression in the lungs altered the phenotypes of alveolar macrophages in terms of the number of alveolar macrophages and surface marker expression at resting state in vivo. Although over-expression of CCL1 in the lungs did not alter the acute inflammatory reaction, it altered the chronic inflammatory response induced by *Mycobacterium*, perhaps through enhancing ER stress. To the best of our knowledge, this study demonstrates novel in vivo roles for CCL1 in adaptive immunity such as granuloma formation.

## Abbreviations

AM: Alveolar macrophage
BAL: Bronchoalveolar lavage
BCG: Bacille de Calmette et Guérin
CCL1: C-C motif ligand 1
COPD: Chronic obstructive pulmonary disease
ER: Endoplasmic reticulum
Ern1: Endoplasmic reticulum to nucleus signaling 1
FSC: Forward scatter
Ig: Immunoglobulin
IRE1a: Inositol-requiring enzyme-1a
LPS: Lipopolysaccharide
SP-C: Surfactant protein-C
SSC: Side scatter
Tg: Transgenic
WT: Wild type
